# Supplementary material for: Risk of Ischemic Stroke Associated with Calcium Supplements and Interaction with Oral Bisphosphonates: A Nested Case-Control Study
Source: J Clin Med. 2023 Aug 14;12(16):5294. doi: 10.3390/jcm12165294 (PMC10455805; doi:10.3390/jcm12165294)
Supplement: Supplementary file 1 [file jcm-12-05294-s001.zip › jcm-2553234-supplementary.pdf]

Journal of
Clinical Medicine

Article ***Supplementary Material***

1. Supplementary Methods

1.1. Data Source.

BIFAP (Base de datos para la Investigación Farmacoepidemiológica en el Ámbito Público) is a healthcare database of primary care medical records for pharmacoepidemiological studies, managed by the Spanish Agency of Medicines and Medical Devices (AEMPS), with the collaboration of 10 Spanish regions (out of 17). The BIFAP population is representative of the population receiving medical care in Spain [1]. BIFAP contains pseudonymized health records of patients attended by primary care physicians (PCPs). Among this information, we can find demographic data, medical diagnostics, vaccinations, free test comments, laboratory tests, complementary explorations and drug prescriptions (indication, product name, dosage, date of prescription, and duration of treatment). Depending on the region, clinical outcomes are recorded using the International Classification of Primary Care, version 2 (ICPC-2) or the International Classification of Diseases, version 9, Clinical Modification (ICD-9-CM). This study was carried out using the 2016 version, which included 7.6 million patients with an average of 5.1 years of follow-up per subject (a total of 38.8 million person-years).

1.2. Stroke case validation procedures

In order to adequately perform pharmacoepidemiological studies in BIFAP, the outcome of interest recorded in the data source is validated. The BIFAP database makes predefined case-finding algorithms (CFA) available to researchers for clinical events based on the appropriate selection of codes within ICPC-BIFAP or ICD-9 codes. The validation procedures for the predefined stroke CFA included the following steps:

1. Establish case-defining criteria and detection of potential stroke cases through CFA.

2. Manual review of the electronic medical record -including clinical notes-in a random sample of 1000 potential stroke cases to confirm or rule out the diagnosis according to the prespecified criteria. This review also validated the date of the event.

3. Estimating the predefined stroke CFA's positive predictive value (PPV) to identify valid incident ischemic strokes.

4. Stroke CFA refinement strategies: Using natural language processing (NLP) techniques to increase the PPV in those subsets with PPV lower than 80%.

Predefined CFA for ischemic, hemorrhagic, or unspecified stroke are available in the BIFAP database. After excluding hemorrhagic strokes, we identified by CFA 24094 ischemic strokes and 15868 unspecified strokes identified, with a total of 39962 cases. A sample of 1000 cases was randomly selected for validation purposes. The sample's electronic healthcare records (EHRs) were reviewed manually, regardless of drug exposure, independently by two investigators (SRM and DBH) for additional information to confirm that it was a real case of incident stroke. The entire research group resolved discrepancies. Patients were classified according to pre-specified criteria in:

- Valid cases: when additional evidence was found in the EHRs confirming the diagnosis;

- Cases with insufficient information: when there was no additional information in the EHRs to support or rule out the diagnosis of stroke;

- No cases: when the available information allowed the diagnosis of an incident stroke to be ruled out.

The review of the random sample of 1000 potential stroke cases yielded the following results: 641 valid cases, 168 insufficient information, and 191 non-cases.

1.2.1. Refinement procedures to increase the PPV of the predefined ischemic stroke CFA.

Natural language processing techniques were applied to identify the following:

a) Semantic patterns in the clinical notes with a high probability of being in the clinical records of valid stroke cases.

b) Semantic patterns in the clinical notes with a high probability of being in the clinical records of non-cases and a low probability of being in the clinical records of valid cases.

Subsequently, the semantic patterns identified in (a) were added to the predefined stroke CFA as additional criteria for stroke case selection, and the semantic patterns identified in (b) were used to consider them as non-cases.

With this refinement, the resulting PPV in the random sample increased to 87.1%. Consequently, the refined incident stroke CFA was applied to all potential cases of ischemic stroke. A total of 14374 cases were included in the case-control study.

1.2.2. Identification of the main pathophysiological subtype among ischemic stroke cases.

In this step, we performed a second validation to identify the most probable pathophysiological subtype of IS (cardioembolic, non-cardioembolic)[2]. In this validation process, cases of stroke due to rare causes (vascular dissections, drug addiction, vasculitis) were sought and excluded.

The following criteria were applied to identify cases of cardioembolic stroke:

- Cardioembolic text criterion: The word "cardioembolic" or other related terms were searched for in-text comments associated with the stroke diagnosis.

- Atrial fibrillation criterion: Patients diagnosed with atrial fibrillation prior to stroke or within 3 months of stroke diagnosis.

- Oral anticoagulants drug use criterion: patients on oral anticoagulant therapy at the time of stroke diagnosis (or up to 3 months prior to diagnosis) or who initiated anticoagulant therapy within 3 months of stroke diagnosis.

The following additional criteria were used when at least one of the above was met:

- Antiarrhythmic drug use criterion: patients on antiarrhythmic drug treatment (class IC and III) at the time of stroke diagnosis (or up to 3 months prior to diagnosis) or initiating treatment within a 3-month window of stroke diagnosis.

- Mitral valve prosthesis/stenosis criterion: patients with a record of mechanical valve prosthesis or mitral stenosis prior to stroke diagnosis or up to 3 months after stroke.

Regardless of the above criteria, all patients who had text strings in the commentary associated with the stroke diagnosis compatible with the words atherothrombotic or lacunar (including related terms) were classified as probable non-cardioembolic ischemic stroke.

Patients with a free text describing a vascular dissection, cocaine abuse, and vasculitis as the probable cause of the stroke were identified in a distinct category and excluded from the analyses.

2. Supplementary Figures and Tables

2.1. Supplementary figures.

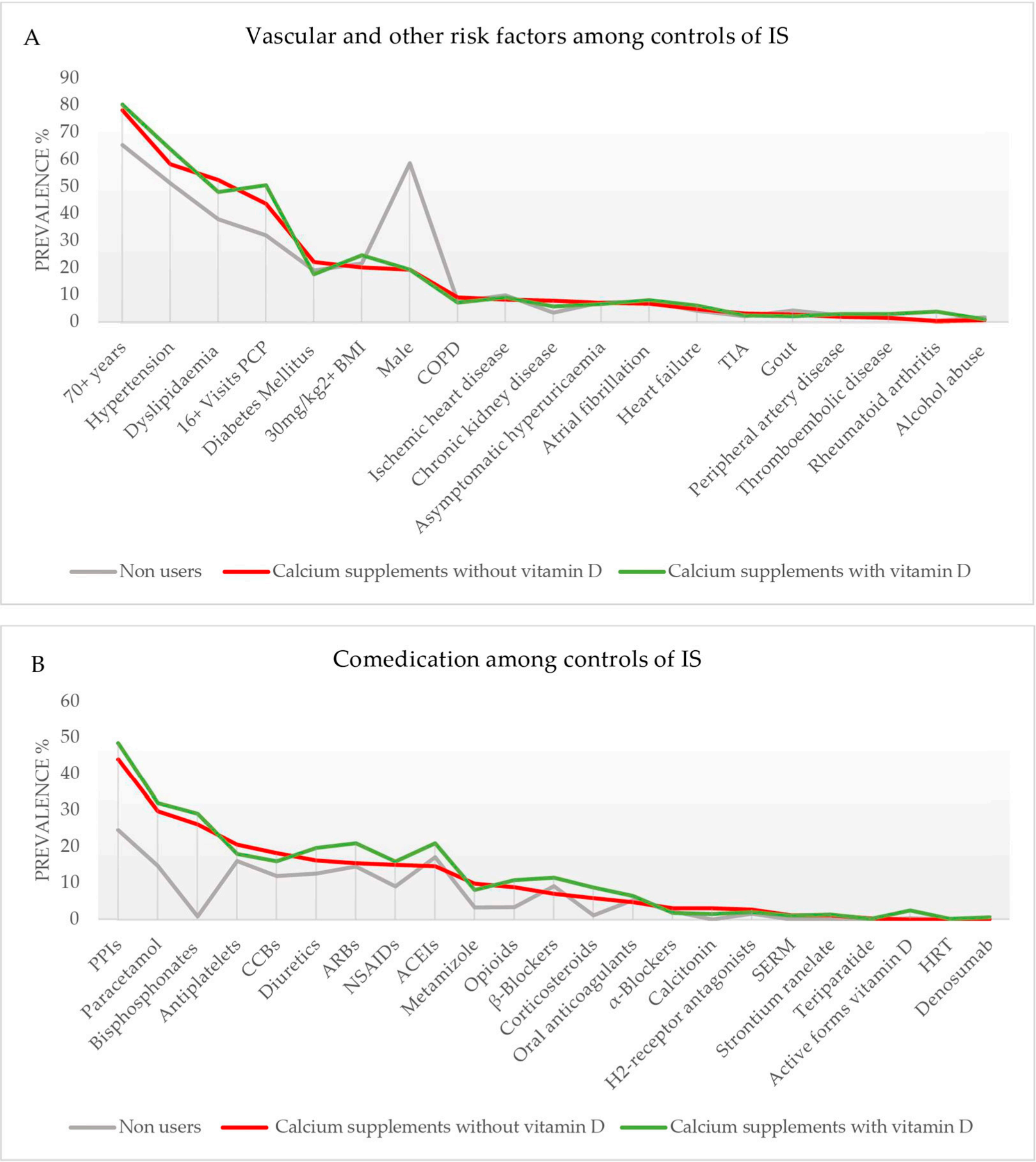

Figure S1. Vascular and other risk factors (A), and comedication (B) among current users of calcium supplementation with or without vitamin D and non-users. All of them from the control series of IS (overall). Abbreviations: ACEIs: Angiotensin Converting Enzyme Inhibitors; ARBs: Angiotensin II Receptor Blockers; BMI: Body Mass Index; CCBs: Calcium Channel Blockers; COPD: Chronic Obstructive Pulmonary Disease; HRT: Hormonal replacement therapy; IS: Ischemic stroke; NSAIDs: Nonsteroidal anti-inflammatory drugs; PCP: Primary Care Physician; PPIs: Proton pump inhibitors; SERM: selective estrogen receptor modulators; TIA: Transient ischemic attack. Diabetes Mellitus: recorded as such, and/or when patients were using glucose-lowering drugs. Dyslipidemia: recorded as such, and/or when patients were using lipid-lowering drugs. Ischemic heart disease: include acute myocardial infarction and angina pectoris (recorded as such and/or use of nitrates)**.** Hormone replacement therapy: including tibolone.

Table S1. Distribution of vascular and other risk factors and comedication in new users of CaM or CaD compared to non-users, among control series of the IS cases (overall).

	CaM users n (%)	CaD users n (%)	Non users n (%)	Proportion´s difference (CaM and non-users)	Proportion´s difference (CaD and non-users)
70+ years	78.2	80.2	65.4	12.8	14.9
Hypertension	58.3	63.8	51.2	7.06	12.5
Dyslipidemia	52.4	48.0	38.0	14.4	10.03
16+ Visits PCP	43.7	50.5	32.0	11.6	18.5
Diabetes Mellitus	22.2	17.6	19.1	3.1	-1.46
30mg/kg^2+ BMI	20.2	24.7	21.6	-1.44	3.01
Male	19.4	19.4	58.7	-39,3	-39.3
COPD	9.13	7.15	7.61	1.52	-0.46
Ischemic heart disease	8.33	9.09	9.96	-1.63	-0.87
Chronic kidney disease	7.94	5.83	3.49	4.45	2.34
Asymptomatic hyperuricemia	7.14	6.69	7.29	-0.15	-0.6
Atrial fibrillation	6.75	8.10	7.41	-0.66	0.69
Heart failure	4.76	6.11	4.19	0.57	1.92
TIA	3.17	2.49	2.13	1.04	0.36
Gout	2.78	2.17	4.26	-1.48	-2.09
Peripheral artery disease	1.98	2.94	2.51	-0.53	0.43
Thromboembolic disease	1.59	2.94	1.50	0.09	1.44
Rheumatoid arthritis	0.40	3.84	0.53	-0.13	3.31
Alcohol abuse	0.79	1.00	1.72	-0.93	-0.72
Current use of:					
PPIs	44.1	48.5	24.6	19.5	23.9
Paracetamol	29.8	32.0	14.8	15.0	17.2
Bisphosphonates	26.2	29.1	0.86	25.3	28.2
Antiplatelets	20.6	18.1	16.1	4.5	1.95
CCBs	18.3	16.1	12	6.3	4.06
Diuretics	16.3	19.7	12.7	3.6	7.02
ARBs	15.5	20.1	14.6	0.9	6.4
NSAIDs	15.1	16.0	9.12	5.9	6.9
ACEIs	14.7	21.0	17.2	-2.5	3.8
Metamizole	9.9	8.2	3.4	6.5	4.8
Opioids	8.9	10.9	3.5	5.4	7.4
β-Blockers	7.1	11.5	9.2	-2.1	2.3
Corticosteroids	5.9	8.9	1.2	4.8	7.7
Oral anticoagulants	4.8	6.5	5.4	-0.65	1.1
α-Blockers	3.2	1.9	2.3	0.9	-0.4
Calcitonin	3.2	1.6	0.03	3.14	1.55
H2-receptor antagonists	2.8	2.1	1.7	1.11	0.41
SERM	1.19	1.13	0.1	1.1	1.03

Table S1. (Continued) Distribution of vascular and other risk factors and comedication in new users of CaM or CaD compared to non-users, among control series of the IS cases (overall).

Strontium ranelate	1.2	1.4	0.07	1.12	1.33
Teriparatide	0.4	0.23	0.02	0.38	0.21
Active forms vitamin D	0	2.53	0.34	-0.34	2.19
HRT	0	0.23	0.1	-0.1	0.13
Denosumab	0	0.63	0.01	-0.01	0.2

Abbreviations: ACEIs: Angiotensin Converting Enzyme Inhibitors; ARBs: Angiotensin II Receptor Blockers; BMI: Body Mass Index; CaD: calcium supplements with vitamin D; CaM: calcium supplements without vitamin D; CCBs: Calcium Channel Blockers; COPD: Chronic Obstructive Pulmonary Disease; HRT: Hormonal replacement therapy; NSAIDs: Nonsteroidal anti-inflammatory drugs; PCP: Primary Care Physician; PPIs: Proton pump inhibitors; SERM: selective estrogen receptor modulators; TIA: Transient ischemic attack.

Table S2. Characteristics of ischemic stroke cases and controls.

	**CASES n=13,267**	**CONTROLS n=61,378**	**Unadjusted OR[a] (95% CI)**
Age, mean (SD), years	74.3 (±12.5)	73.9 (±12.6)	NA
Men, (%)	7021 (52.92)	34,491 (56.19)	NA
Number of visits to PCP, (%)			
<6	2416 (18.21)	18,061 (29.43)	1 (ref.)
6-15	4922 (37.10)	23,073 (37.59)	1.66 (1.57–1.75)
16-24	3060 (23.06)	11,042 (17.99)	2.26 (2.12–2.40)
>24	2869 (21.63)	9202 (14.99)	2.64 (2.48–2.82)
BMI, No. (%), kg.m^{-2}			
<25	1827 (13.77)	8090 (13.18)	1 (ref.)
25-29.9	3915 (29.51)	17,860 (29.10)	0.98 (0.92–1.04)
30-34.9	2371 (17.87)	10,138 (16.52)	1.04 (0.97–1.11)
35-39.9	683 (5.15)	2597 (4.23)	1.15 (1.04–1.27)
≥40	228 (1.72)	677 (1.10)	1.47 (1.25–1.73)
Unknown	4243 (31.98)	22,016 (35.87)	0.86 (0.81–0.91)
Smoking, (%)			
Non-smoker	4291 (32.34)	19,427 (31.65)	1 (ref)
Current smoker	2191 (16.51)	7437 (12.12)	1.50 (1.41–1.60)
Past smoker	930 (7.01)	3494 (5.69)	1.34 (1.24–1.46)
Unknown	5855 (44.13)	31,020 (50.54)	0.87 (0.84–0.91)
Alcohol abuse[c]	411 (3.10)	1018 (1.66)	2.02 (1.80–2.27)
Diabetes[d]	3869 (29.16)	11,707 (19.07)	1.76 (1.69–1.84)
Hyperuricemia:			
Asymptomatic	1062 (8.00)	4453 (7.26)	1.14 (1.07–1.23)
Gout	675 (5.09)	2561 (4.17)	1.30 (1.19–1.42)
Hypertension	8244 (62.14)	31,918 (52.00)	1.54 (1.48–1.61)
Dyslipidemia	5841 (44.03)	23,776 (38.74)	1.25 (1.20–1.30)
Peripheral artery disease	672 (5.07)	1550 (2.53)	2.14 (1.95–2.35)
Ischemic heart disease:			
Acute myocardial infarction	820 (6.18)	2272 (3.70)	1.84 (1.69–2.00)
Angina pectoris[e]	1182 (8.91)	3857 (6.28)	1.52 (1.42–1.63)
TIA	737 (5.56)	1330 (2.17)	2.66 (2.42–2.92)
Atrial fibrillation	1951 (14.71)	4594 (7.48)	2.17 (2.04–2.30)

**Table S2. (Continued)** Characteristics of ischemic stroke cases and controls.			
Thromboembolic disease	284 (2.14)	967 (1.58)	1.36 (1.19–1.55)
Heart failure	978 (7.37)	2653 (4.32)	1.76 (1.63–1.90)
Chronic renal failure	731 (5.51)	2248 (3.66)	1.54 (1.41–1.68)
Rheumatoid arthritis	79 (0.60)	415 (0.68)	0.87 (0.68–1.10)
COPD	1151 (8.68)	4659 (7.59)	1.21 (1.13–1.29)
**Current use of:**			
Antiplatelet drugs	3524 (26.56)	9971 (16.25)	2.15 (2.05–2.25)
NSAIDs	1190 (8.97)	5818 (9.48)	0.99 (0.92–1.06)
Oral anticoagulants drugs	1042 (7.85)	3366 (5.48)	1.52 (1.41–1.64)
Paracetamol (acetaminophen)	2161 (16.29)	9661 (15.74)	1.14 (1.08–1.21)
Metamizole	627 (4.73)	2247 (3.66)	1.38 (1.26–1.52)
Opioids	625 (4.71)	2389 (3.89)	1.23 (1.12–1.35)
Proton pump inhibitors	4233 (31.91)	15,923 (25.94)	1.44 (1.37–1.51)
H2-receptor antagonists	310 (2.34)	1045 (1.70)	1.40 (1.23–1.59)
Corticosteroids	268 (2.02)	943 (1.54)	1.35 (1.17–1.55)
ACEIs	2805 (21.14)	10,625 (17.31)	1.40 (1.34–1.47)
ARBs	2331 (17.57)	9198 (14.99)	1.26 (1.19–1.32)
Calcium channel blockers	2052 (15.47)	7461 (12.16)	1.42 (1.34–1.50)
β-Blockers	2033 (15.32)	5733 (9.34)	1.85 (1.75–1.96)
α-Blockers	345 (2.60)	1390 (2.26)	1.19 (1.06–1.35)
Diuretics	2367 (17.84)	7995 (13.03)	1.56 (1.48–1.65)
Active forms of vitamin D	73 (0.55)	279 (0.45)	1.19 (0.92–1.55)
Bisphosphonates	299 (2.25)	1,324 (2.16)	0.98 (0.86–1.11)
Hormonal replacement therapy [f]	11 (0.08)	65 (0.11)	0.76 (0.40–1.46)
SERM	17 (0.13)	100 (0.16)	0.72 (0.43–1.21)
Strontium ranelate	24 (0.18)	78 (0.13)	1.28 (0.80–2.03)
Calcitonin	8 (0.06)	62 (0.10)	0.59 (0.28–1.23)
Denosumab	6 (0.05)	24 (0.04)	1.06 (0.43–2.60)
Teriparatide	9 (0.07)	33 (0.05)	1.16 (0.55–2.45)

Abbreviations: ACEIs: Angiotensin Converting Enzyme Inhibitors; ARBs: Angiotensin II Receptor Blockers; BMI: Body Mass Index; COPD: Chronic Obstructive Pulmonary Disease; NSAIDs: Nonsteroidal anti-inflammatory drugs; PCP: Primary Care Physician; SERM: selective estrogen receptor modulators; TIA: Transient ischemic attack. [a] Model adjusted only for matching variables (age, sex and index date). The category of reference was "no presence of the disease or risk factor". For drugs the category of reference was "non-use". [b] When the general practitioner recorded an excessive consumption of alcohol. [c] Recorded as such or when patients were using glucose-lowering drugs. [d] Recorded as such or when patients were using lipid-lowering drugs. [e] Recorded as such and/or use of nitrates. [f] Including tibolone.

Table S3. Risk of ischemic stroke associated with the use of calcium supplements applying multiple imputation by chained equation models.

Overall IS	Cases (%) N=13,267	Controls (%) N=61,378	Unadjusted OR* (95% CI)	Adjusted OR† (95% CI)
Non-users	12,225 (92.15)	57,434 (93.57)	1 (Ref.)	1 (Ref.)
Recency of use				
• Current users of CaM	77 (0.58)	252 (0.41)	1.36 (1.05–1.76)	1.26 (0.96–1.66)
• Current users of CaD	543 (4.09)	2211 (3.60)	1.09 (0.99–1.21)	1.03 (0.92–1.16)
• Past users of CaM/CaD	422 (3.18)	1481 (2.41)	1.28 (1.14–1.43)	1.14 (1.00–1.29)
Duration				
• Among current users of CaM				
≤ 1year	50 (0.38)	186 (0.30)	1.19 (0.87–1.63)	1.10 (0.79–1.53)
> 1 year	27 (0.20)	66 (0.11)	1.84 (1.17–2.88)	1.76 (1.10–2.84)
• Among current users of CaD				
≤ 1year	368 (2.70)	1571 (2.56)	1.02 (0.91–1.15)	0.97 (0.85–1.11)
> 1 year	185 (1.39)	640 (1.04)	1.28 (1.08–1.51)	1.19 (0.99–1.44)
Daily dose of calcium				
• Among current users of CaM				
Low dose (<1000mg/d)	41 (0.31)	145 (0.24)	1.24 (0.87–1.76)	1.23 (0.85–1.78)
High dose (≥ 1000mg/d)	23 (0.17)	58 (0.09)	1.81 (1.12–2.95)	1.39 (0.83–2.33)
Unknown	13 (0.10)	49 (0.08)	1.19 (0.64–2.19)	1.18 (0.63–2.21)
• Among current users of CaD				
Low dose (<1000mg/d)	216 (1.63)	1022 (1.67)	0.93 (0.80–1.08)	0.87 (0.74–1.03)
High dose (≥ 1000mg/d)	246 (1.85)	862 (1.40)	1.28 (1.11–1.48)	1.20 (1.02–1.41)
Unknown	81 (0.61)	327 (0.53)	1.12 (0.87–1.43)	1.09 (0.84–1.42)

*Adjusted only for matching factors (age, sex and calendar year). †Adjusted for matching factors (age, sex and calendar year) and the potential confounding factors shown in table 1. Except atrial fibrillation and oral anticoagulants drugs.

Table S4. Risk of cardioembolic ischemic stroke associated with the use of calcium supplements applying multiple imputation by chained equation models.

Cardioembolic stroke	Cases (%) N=4400	Controls (%) N=20,147	Unadjusted OR* (95% CI)	Adjusted OR† (95% CI)
Non-users	3995 (90.80)	18,687 (92.75)	1 (Ref.)	1 (Ref.)
Recency of use				
• Current users of CaM	34 (0.77)	90 (0.45)	1.67 (1.12–2.49)	1.87 (1.21–2.90)
• Current users of CaD	224 (5.09)	788 (3.91)	1.27 (1.09–1.49)	1.08 (0.89–1.32)
• Past users of CaM/CaD	147 (3.34)	582 (2.89)	1.14 (0.94–1.37)	0.93 (0.75–1.17)
Duration				
• Among current users of CaM				
≤ 1year	21 (0.48)	70 (0.35)	1.32 (0.80–2.15)	1.58 (0.93–2.69)
> 1 year	13 (0.30)	20 (0.10)	2.95 (1.46–5.96)	2.76 (1.27–6.02)
• Among current users of CaD				
≤ 1year	159 (3.61)	531 (2.64)	135 (1.12–1.62)	1.18 (0.95–1.47)
> 1 year	65 (1.48)	257 (1.28)	1.12 (0.85–1.48)	0.88 (0.64–1.22)
Daily dose of calcium				
• Among current users of CaM				
Low dose (<1000mg/d)	21 (0.48)	50 (0.25)	1.80 (1.08–3.01)	2.24 (1.27–3.93)
High dose (≥ 1000mg/d)	9 (0.20)	20 (0.10)	2.09 (0.95–4.61)	1.64 (0.70–3.84)
Unknown	4 (0.09)	20 (0.10)	0.89 (0.30–2.63)	1.22 (0.40–3.73)
• Among current users of CaD				
Low dose (<1000mg/d)	90 (2.05)	375 (1.86)	1.06 (0.83–1.34)	0.91 (0.69–1.19)
High dose (≥ 1000mg/d)	101 (2.30)	299 (1.48)	1.53 (1.22–1.93)	1.31 (1.00–1.72)
Unknown	33 (0.75)	114 (0.57)	1.33 (0.90–1.96)	1.09 (0.70–1.70)

*Adjusted only for matching factors (age, sex and calendar year). †Adjusted for matching factors (age, sex and calendar year) and the potential confounding factors shown in table 1. Except atrial fibrillation and oral anticoagulants drugs.

Table S5. Non-cardioembolic ischemic stroke associated with use of calcium supplements applying multiple imputation by chained equation models.

Non-cardioembolic stroke	Cases (%) N=8867	Controls (%) N=41,231	Unadjusted OR* (95% CI)	Adjusted OR† (95% CI)
Non-users	8230 (92.82)	38,747 (93.98)	1 (Ref.)	1 (Ref.)
Recency of use				
• Current users of CaM	43 (0.48)	162 (0.39)	1.19 (0.85–1.67)	1.08 (0.76–1.55)
• Current users of CaD	319 (3.60)	1423 (3.45)	0.99 (0.88–1.13)	0.99 (0.86–1.15)
• Past users of CaM/CaD	275 (3.10)	899 (2.18)	1.37 (1.19–1.58)	1.26 (1.07–1.48)
Duration				
• Among current users of CaM				
≤ 1year	29 (0.33)	116 (0.28)	1.12 (0.74–1.69)	1.00 (0.65–1.53)
> 1 year	14 (0.16)	46 (0.11)	1.36 (0.74–2.48)	1.37 (0.73–2.57)
• Among current users of CaD				
≤ 1year	199 (2.24)	1040 (2.52)	0.85 (0.73–1.00)	0.86 (0.72–1.02)
> 1 year	120 (1.35)	383 (0.93)	1.38 (1.12–1.70)	1.40 (1.11–1.78)
Daily dose of calcium				
• Among current users of CaM				
Low dose (<1000mg/d)	20 (0.23)	95 (0.23)	0.94 (0.58–1.52)	0.90 (0.54–1.50)
High dose (≥ 1000mg/d)	14 (0.16)	38 (0.09)	1.67 (0.90–3.09)	1.35 (0.70–2.60)
Unknown	9 (0.10)	29 (0.07)	1.39 (0.65–2.94)	1.28 (0.59–2.79)
• Among current users of CaD				
Low dose (<1000mg/d)	126 (1.42)	647 (1.57)	0.86 (0.71–1.05)	0.84 (0.68–1.04)
High dose (≥ 1000mg/d)	145 (1.64)	563 (1.37)	1.14 (0.95–1.38)	1.14 (0.93–1.40)
Unknown	48 (0.54)	213 (0.52)	1.01 (0.74–1.38)	1.08 (0.78–1.51)

*Adjusted only for matching factors (age, sex and calendar year).†Adjusted for matching factors (age, sex and calendar year) and the potential confounding factors shown in table 1. Except atrial fibrillation and oral anticoagulants drugs.

Supplementary Materials: Figure S1: Vascular and other risk factors (A), and comedication (B) among current users of calcium supplementation with or without vitamin D and non-users. All of them from the control series of IS (overall).; Table S1: Distribution of vascular and other risk factors and comedication in new users of CaM or CaD compared to non-users, among control series of the IS cases (overall);Table S2: Characteristics of ischemic stroke cases and controls; Table S3: Risk of ischemic stroke associated with the use of calcium supplements applying multiple imputation by chained equation models; Table S4: Risk of cardioembolic ischemic stroke associated with the use of calcium supplements applying multiple imputation by chained equation models; Table S5: Non-cardioembolic ischemic stroke associated with use of calcium supplements applying multiple imputation by chained equation models.

References

1. Maciá-Martínez M, Gil M, Huerta C, Martín-Merino E, Álvarez A, Bryant V, et al. *Base de Datos para la Investigación Farmacoepidemiológica en Atención Primaria* (BIFAP): A data resource for pharmacoepidemiology in Spain. Pharmacoepidemiol Drug Saf. 2020, 29, 1236-1245. [CrossRef] [PubMed]

2. Alqdwah-Fattouh R, Rodríguez-Martín S, Barreira-Hernández D, Izquierdo-Esteban L, Gil M, González-Bermejo D, et al. Selective Serotonin Reuptake Inhibitors and Risk of Noncardioembolic Ischemic Stroke: A Nested Case-Control Study. Stroke. 2022, 53, 1560-1569. [CrossRef] [PubMed]

Disclaimer/Publisher's Note: The statements, opinions and data contained in all publications are solely those of the individual author(s) and contributor(s) and not of MDPI and/or the editor(s). MDPI and/or the editor(s) disclaim responsibility for any injury to people or property resulting from any ideas, methods, instructions or products referred to in the content.
